# Supplementary material for: Phytochemical Composition and Antioxidant Activity of a Viscum album Mother Tincture
Source: Plants (Basel). 2025 Sep 4;14(17):2762. doi: 10.3390/plants14172762 (PMC12430634; doi:10.3390/plants14172762)
Supplement: Supplementary file 1 [file plants-14-02762-s001.zip › plants-3733472-supplementary.pdf]

Supplementary Information

Table S1. HEAVY and light viscotoxins peptides. The table shows the transitions and collision energies followed for the peptides of interest.

Protein	Precursor.m/z	Collision energy	Product.m/z	Heavy Peptide
**Viscotoxin A**	592.28	21	744.33<br>584.30<br>487.24<br>386.19<br>343.20<br>440.25<br>479.71	LTGAP**C**PT**C**A**K**
**Viscotoxin A1**	518.21	18	628.33<br>531.28<br>444.24<br>343.20<br>408.10<br>505.15<br>592.19	S**CC**PSTTG**R**
**Viscotoxin A2**	559.76	19	730.36<br>659.33<br>572.30<br>459.21<br>389.15<br>460.19	EV**C**ASLSG**CK**
**Viscotoxin A3**	903.04	31	928.44<br>983.40<br>902.35<br>933.86<br>990.88	FY**C**TLG**C**ESSQ**C**ATNSNGDAEAV**R**
	590.32	21	740.40<br>584.30<br>487.24<br>386.19<br>483.26<br>343.20<br>440.25<br>596.35	LTGAPRPT**C**A**K**
	716.85	25	874.39<br>714.35<br>617.30<br>530.27<br>415.24<br>371.23<br>458.26<br>559.31	IISGST**C**PSDYP**K**
**Viscotoxin B**	642.97	22	769.41<br>670.34<br>647.32<br>598.79<br>319.11<br>432.19<br>533.24	**C**ASITTPPNSEVDAEAV**R**
			386.20<br>329.18<br>333.16<br>420.19	
			561.23<br>446.21<br>345.16<br>506.22<br>620.27	

Protein	Precursor.m/z	Collision energy	Product.m/z	Light Peptide
**Viscotoxin A**	588,28	21	736,31<br>576,28<br>479,23<br>378,18<br>343,20<br>440,25<br>958,41	LTGAPCPTCAK
**Viscotoxin A1**	526,72	18	645,33<br>548,28<br>434,24<br>408,10<br>505,15	SCCPNTTGR
**Viscotoxin A2**	555,75	19	722,35<br>651,31<br>564,28<br>451,20<br>389,15<br>460,19	EVCASLSGCK
**Viscotoxin A3**	899,70	31	918,43<br>660,33<br>978,39<br>602,78<br>567,26	FYCTLGCESSQCATNSNGDAEAVR
	586,32	21	732,38<br>576,28<br>479,23<br>378,18<br>479,25<br>343,20<br>440,25<br>596,35	LTGAPRPTCAK
	712,84	25	866,37<br>706,34<br>609,29<br>522,26<br>407,23<br>371,23<br>458,26<br>559,31	IISGSTCPSDYPK
**Viscotoxin B**	441,69	15	651,31<br>564,28<br>451,20<br>364,16<br>319,11<br>432,19	CASLSGCK
**Viscotoxin-C1**	310,67	10	507,25<br>406,20<br>349,18<br>359,19<br>446,22	LTGSSR
**Viscotoxin-1-PS**	471,21	16	713,80<br>550,73<br>436,20<br>335,15<br>506,22<br>607,27	BIYBTCR

Table S2. The transitions and collision energies followed for the polyphenols are reported

precursor	product	Dwell	Analyte	DP	CE
285.000	151.000	50.0	Kaempferol	-86.000	-28.000
285.000	92.800	50.0	Kaempferol	-86.000	-28.000
285.000	133.000	50.0	Kaempferol	-60.000	-28.000
242.800	158.900	50.0	Piceatannol	-36.000	-38.000
242.800	201.000	50.0	Piceatannol	-36.000	-38.000
431.000	268.700	50.0	Genistin	-86.000	-18.000
431.000	132.800	50.0	Genistin	-86.000	-18.000
609.000	301.000	50.0	Rutin	-60.000	-30.000
609.000	299.800	50.0	Rutin	-61.000	-50.000
609.000	270.900	50.0	Rutin	-61.000	-50.000
579.100	270.800	50.0	Naringin	-96.000	-56.000
579.100	150.800	50.0	Naringin	-96.000	-56.000
227.000	185.000	50.0	Resveratrol	-96.000	-32.000
227.000	143.000	50.0	Resveratrol	-96.000	-32.000
316.800	150.800	50.0	Myricetin	-56.000	-36.000
316.800	178.900	50.0	Myricetin	-56.000	-36.000
286.800	150.700	50.0	Eriodictyol	-31.000	-22.000
286.800	134.900	50.0	Eriodictyol	-31.000	-22.000
356.900	82.900	50.0	Matairesinol	-31.000	-36.000
356.900	137.000	50.0	Matairesinol	-31.000	-36.000
301.000	252.900	50.0	Enterodiol	-56.000	-26.000
301.000	106.000	50.0	Enterodiol	-56.000	-26.000
300.900	150.900	50.0	Quercetin	-56.000	-32.000
300.900	178.600	50.0	Quercetin	-56.000	-32.000
284.900	174.900	50.0	Cyanidin	-61.000	-32.000
284.900	240.800	50.0	Cyanidin	-61.000	-32.000
285.000	132.900	50.0	Luteolin	-61.000	-46.000
285.000	150.900	50.0	Luteolin	-61.000	-46.000
285.000	217.000	50.0	Luteolin	-60.000	-46.000
285.000	199.000	50.0	Luteolin	-60.000	-46.000
296.900	106.900	50.0	Enterolactone	-46.000	-46.000
296.900	253.000	50.0	Enterolactone	-46.000	-46.000
288.800	245.000	50.0	Catechin	-71.000	-22.000
288.800	108.900	50.0	Catechin	-71.000	-22.000
166.600	123.100	50.0	Vanillic acid	-61.000	-18.000
166.600	107.800	50.0	Vanillic acid	-61.000	-18.000
178.700	107.200	50.0	Caffeic acid	-21.000	-22.000
288.800	244.800	50.0	Epicatechin	-76.000	-22.000
288.800	109.100	50.0	Epicatechin	-76.000	-22.000
300.900	256.700	50.0	Delphinidin	-51.000	-20.000
300.900	190.800	50.0	Delphinidin	-51.000	-20.000
268.800	240.900	50.0	Pelargonidin	-61.000	-26.000
268.800	200.700	50.0	Pelargonidin	-61.000	-26.000

163.000	162.000	50.0	transpCoumaric acid	-35.000	-20.000
163.000	119.000	50.0	transpCoumaric acid	-35.000	-20.000
300.800	163.900	50.0	Haesperetin	-51.000	-34.000
300.800	150.900	50.0	Haesperetin	-51.000	-34.000
268.600	132.900	50.0	Genistein	-101.000	-44.000
268.600	159.000	50.0	Genistein	-101.000	-44.000
267.000	210.900	50.0	Coumestrol	-81.000	-38.000
267.000	238.800	50.0	Coumestrol	-80.000	-44.000
268.800	116.900	50.0	Apigenin	-46.000	-44.000
268.800	150.800	50.0	Apigenin	-45.000	-30.000
266.800	251.700	50.0	Formonetin	-91.000	-30.000
266.800	222.800	50.0	Formonetin	-90.000	-28.000
282.800	267.500	50.0	Glycitein	-101.000	-28.000
282.800	240.000	50.0	Glycitein	-100.000	-32.000
282.900	267.800	50.0	Biochanin A	-76.000	-28.000
282.900	238.800	50.0	Biochanin A	-76.000	-28.000
254.900	239.900	50.0	Pterosilbene	-31.000	-28.000
449.000	431.000	50.0	luteolin6cglucoside (ISOORIENTIN)	-60.000	-30.000
449.000	285.000	50.0	luteolin6cglucoside (ISOORIENTIN)	-60.000	-30.000
449.000	377.000	50.0	luteolin6cglucoside (ISOORIENTIN)	-60.000	-30.000
431.000	311.000	50.0	Apigenin8cglucoside (VITEXIN)	-55.000	-30.000
431.000	269.000	50.0	Apigenin8cglucoside (VITEXIN)	-55.000	-30.000
431.000	341.000	50.0	Apigenin8cglucoside (VITEXIN)	-55.000	-30.000
464.000	301.000	50.0	Quercetin3Ogalactoside	-60.000	-38.000
464.000	463.000	50.0	Quercetin3Ogalactoside	-60.000	-38.000
594.000	593.000	50.0	kaempferol3Orutinoside	-65.000	-45.000
594.000	285.000	50.0	kaempferol3Orutinoside	-65.000	-45.000
316.000	300.000	50.0	Isorhamnetin	-60.000	-30.000
316.000	151.000	50.0	Isorhamnetin	-60.000	-30.000
316.000	315.000	50.0	Isorhamnetin	-60.000	-30.000
538.000	537.000	50.0	Amentoflavone	-60.000	-40.000
538.000	375.000	50.0	Amentoflavone	-60.000	-40.000
353.000	191.000	50.0	caffeoylquinic acid	-60.000	-30.000
353.000	179.000	50.0	caffeoylquinic acid	-60.000	-30.000
609.000	463.000	50.0	quercetin rutinoside	-60.000	-30.000
609.000	300.000	50.0	quercetin rutinoside	-60.000	-30.000
505.000	463.000	50.0	Quercetin acetylhexoside	-60.000	-30.000
505.000	445.000	50.0	Quercetin acetylhexoside	-60.000	-30.000
183.000	169.000	50.0	methyl gallate	-60.000	-20.000
183.000	125.000	50.0	methyl gallate	-60.000	-20.000
593.000	425.000	50.0	EGCepicatechin dimer	-60.000	-30.000
197.000	169.000	50.0	ethyl gallate	-60.000	-20.000
197.000	125.000	50.0	ethyl gallate	-60.000	-20.000
729.000	577.000	50.0	Procyanidin dimer gallate	-60.000	-30.000
493.000	317.000	50.0	Myricetin3Oglucuronide	-40.000	-25.000
479.000	317.000	50.0	Myricetin3Oglucoside	-40.000	-25.000

441.000	289.000	50.0	EC3Ogallate	-60.000	-30.000
441.000	169.000	50.0	EC3Ogallate	-60.000	-30.000
389.000	227.000	50.0	cisresveratrol3Oglucoside	-40.000	-25.000
561.000	289.000	50.0	EfisetinidolEC isomer 2	-71.000	-22.000
561.000	273.000	50.0	EfisetinidolEC isomer 3	-71.000	-22.000
745.000	457.000	50.0	EGCEGC gallate	-60.000	-20.000
745.000	169.000	50.0	EGCEGC gallate	-60.000	-20.000
619.000	457.000	50.0	EGC gallate glucoside	-60.000	-20.000
619.000	305.000	50.0	EGC gallate glucoside	-60.000	-20.000
609.000	457.000	50.0	EGC digallate	-60.000	-20.000
609.000	305.000	50.0	EGC digallate	-60.000	-30.000
455.000	289.000	50.0	EC methyl gallate	-60.000	-30.000
455.000	183.000	50.0	EC methyl gallate	-60.000	-30.000
425.000	169.000	50.0	EAfzelechin gallate	-60.000	-30.000
715.000	563.000	50.0	Theaflavin gallate	-60.000	-30.000
715.000	545.000	50.0	Theaflavin gallate	-60.000	-30.000
867.000	715.000	50.0	Theaflavin diglallate	-60.000	-30.000
867.000	563.000	50.0	Theaflavin diglallate	-60.000	-30.000
563.000	545.000	50.0	Theaflavin	-60.000	-30.000
153.100	133.000	50.0	Protocatechuic Acid	-40.000	-20.000
153.100	109.000	50.0	Protocatechuic Acid	-40.000	-20.000
153.100	93.000	50.0	Protocatechuic Acid	-40.000	-20.000
353.300	191.000	50.0	Chlorogenic Acid	-50.000	-20.000
353.300	163.000	50.0	Chlorogenic Acid	-50.000	-20.000
353.300	145.000	50.0	Chlorogenic Acid	-50.000	-20.000
353.300	179.000	50.0	Chlorogenic Acid	-50.000	-30.000
353.300	135.000	50.0	Chlorogenic Acid	-50.000	-30.000
335.300	179.000	50.0	Caffeoyliquinic Acid Lactone	-60.000	-30.000
335.300	161.000	50.0	Caffeoyliquinic Acid Lactone	-60.000	-30.000
193.200	149.000	50.0	Ferulic Acid	-40.000	-20.000
193.200	134.000	50.0	Ferulic Acid	-40.000	-20.000
591.200	283.000	50.0	Linarin	-40.000	-18.000
591.200	447.000	50.0	Linarin	-40.000	-18.000
591.200	420.000	50.0	Linarin	-40.000	-18.000
415.400	295.000	50.0	Puerarin	-40.000	-30.000
415.400	325.000	50.0	Puerarin	-40.000	-30.000
415.400	399.000	50.0	Puerarin	-40.000	-45.000
415.400	381.000	50.0	Puerarin	-40.000	-45.000
299.300	284.000	50.0	Diosmetin	-40.000	-26.000
299.300	153.000	50.0	Diosmetin	-40.000	-26.000
299.300	201.000	50.0	Diosmetin	-40.000	-26.000
299.300	55.000	50.0	Diosmetin	-40.000	-26.000
769.700	768.000	50.0	Typhanoside	-40.000	-35.000
769.700	314.000	50.0	Typhanoside	-40.000	-35.000
447.400	327.000	50.0	Astragalin	-40.000	-35.000
447.400	284.000	50.0	Astragalin	-40.000	-35.000

163.200	147.000	50.0	Coumaric Acid	-40.000	-20.000
163.200	119.000	50.0	Coumaric Acid	-40.000	-20.000
163.200	91.000	50.0	Coumaric Acid	-40.000	-20.000
337.300	191.000	50.0	3PCoumaroylquinic Acid	-40.000	-30.000
337.300	163.000	50.0	3PCoumaroylquinic Acid	-40.000	-30.000
447.300	431.000	50.0	Orientin	-40.000	-30.000
447.300	413.000	50.0	Orientin	-40.000	-30.000
447.300	357.000	50.0	Orientin	-40.000	-30.000
447.300	285.000	50.0	Orientin	-40.000	-30.000
447.400	285.000	50.0	Luteolin7OGlucoside	-60.000	-30.000
447.400	153.000	50.0	Luteolin7OGlucoside	-60.000	-30.000
447.400	60.000	50.0	Luteolin7OGlucoside	-60.000	-30.000
577.500	270.000	50.0	Isorhoifolin	-60.000	-40.000
577.500	269.000	50.0	Isorhoifolin	-60.000	-40.000
577.500	45.000	50.0	Isorhoifolin	-60.000	-40.000
463.400	43.000	50.0	Hyperoside	-60.000	-38.000
463.400	301.000	50.0	Hyperoside	-60.000	-38.000
447.400	301.000	50.0	QuercetinORhamnoside	-60.000	-30.000
447.400	431.000	50.0	QuercetinORhamnoside	-60.000	-30.000
461.900	299.000	50.0	QuercetinOHexoside	-60.000	-30.000
593.500	285.000	50.0	Nicotinflorin	-60.000	-45.000
593.500	257.000	50.0	Nicotinflorin	-60.000	-45.000
593.500	449.000	50.0	Nicotinflorin	-60.000	-45.000
431.100	285.000	50.0	Kaempeferol3ORhamnoside	-60.000	-30.000
271.200	153.000	50.0	Naringenin	-60.000	-30.000
271.200	177.000	50.0	Naringenin	-60.000	-30.000
271.200	119.000	50.0	Naringenin	-60.000	-30.000
271.200	107.000	50.0	Naringenin	-60.000	-30.000
271.200	93.000	50.0	Naringenin	-60.000	-30.000
271.200	83.000	50.0	Naringenin	-60.000	-30.000
433.400	271.000	50.0	Naringenin7OGlucoside	-60.000	-20.000
433.400	295.000	50.0	Naringenin7OGlucoside	-60.000	-20.000
433.400	270.000	50.0	Naringenin7OGlucoside	-60.000	-20.000
579.500	271.000	50.0	Naringenin7ONeohesperidoside	-80.000	-35.000
579.500	459.000	50.0	Naringenin7ONeohesperidoside	-80.000	-35.000
623.500	314.000	50.0	Isorhamentin3ONeohesperidoside	-80.000	-43.000
179.200	67.000	50.0	Theobromine	-45.000	-30.000
179.200	109.000	50.0	Theobromine	-45.000	-30.000
179.200	83.000	50.0	Theobromine	-45.000	-30.000
179.200	69.000	50.0	Theobromine	-45.000	-30.000
179.200	138.000	50.0	Theobromine	-45.000	-30.000
193.200	109.000	50.0	Caffeine	-45.000	-30.000
193.200	123.000	50.0	Caffeine	-45.000	-30.000
193.200	153.000	50.0	Caffeine	-45.000	-30.000
193.200	60.000	50.0	Procynadin B2	-45.000	-22.000
135.200	54.000	50.0	Tetramethyl Pyrazine	-45.000	-22.000

135.200	80.000	50.0	Tetramethyl Pyrazine	-45.000	-22.000
135.200	121.000	50.0	Tetramethyl Pyrazine	-45.000	-22.000
135.200	96.000	50.0	Tetramethyl Pyrazine	-45.000	-22.000
121.200	54.000	50.0	Trimethyl Pyrazine	-45.000	-22.000
121.200	42.000	50.0	Trimethyl Pyrazine	-45.000	-22.000
121.200	39.000	50.0	Trimethyl Pyrazine	-45.000	-22.000
865.800	289.000	50.0	Procynadin C1	-45.000	-22.000
865.800	393.000	50.0	Procynadin C1	-45.000	-22.000
865.800	713.000	50.0	Procynadin C1	-45.000	-22.000
865.800	849.000	50.0	Procynadin C1	-45.000	-22.000
865.800	577.000	50.0	Procynadin C1	-45.000	-22.000
577.500	427.000	50.0	Procynadin B2	-45.000	-22.000
577.500	291.000	50.0	Procynadin B2	-45.000	-22.000
577.500	561.000	50.0	Procynadin B2	-45.000	-22.000
575.500	109.000	50.0	Proanthocyanidin A2	-45.000	-22.000
575.500	139.000	50.0	Proanthocyanidin A2	-45.000	-22.000
575.500	559.000	50.0	Proanthocyanidin A2	-45.000	-22.000
169.000	125.000	50.0	Gallic acid	-64.000	-20.000
169.000	126.000	50.0	Gallic acid	-49.000	-21.000
169.000	106.800	50.0	Gallic acid	-66.000	-18.000

Table S3. The transitions and collision energies followed for vitamins are reported.

	Precursor ion	Product Ion	CE	DP	dwell
Vitamin B1	265.110	80.900	20.000	60	50
Vitamin B1	265.000	122.100	5.000	60	50
Vitamin B3	123.100	80.100	20.000	60	50
Vitamin B3	123.100	53.000	38.000	60	50
Vitamin B5	220.000	90.150	20.000	60	50
Vitamin B5	220.000	71.900	18.000	60	50
Biotin	245.100	227.100	8.000	60	50
Biotin	245.100	123.000	28.000	60	50
Biotin	245.100	97.100	32.000	60	50
Cyanocobalamin	678.600	147.100	40.000	80	50
Cyanocobalamin	678.600	359.200	30.000	80	50
Folic acid	442.180	145.000	65.000	60	50
Folic acid	442.180	117.000	5.000	60	50
Folic acid	442.180	59.200	53.000	60	50
Pyridoxine	170.080	152.100	12.000	60	50
Pyridoxine	170.080	134.000	24.000	60	50
Pyridoxine	170.080	77.000	40.000	60	50
Riboflavin	377.150	243.000	16.000	60	50
Riboflavin	377.150	198.100	44.000	60	50
Riboflavin	377.150	172.000	16.000	60	50
Vitamin C	177.000	141.000	8.000	50	50
Vitamin C	177.000	95.000	12.000	50	50
Vitamin A	269.300	216.000	20.000	70	50
Vitamin A	269.300	91.150	20.000	70	50
Vitamin A	269.300	93.000	25.000	70	50
Vitamin E	432.200	415.100	5.000	70	50
Vitamin E	432.200	135.100	10.000	70	50
Vitamin E	432.200	119.400	15.000	70	50

Table S4. The transitions and collision energies followed for amino acids are reported.

analyte	precursor m/Z	product m/z	CE	DP	dwell
gly	76,1	30	10	60	50
ala	90,1	44	10	60	50
ser	106,1	60	10	60	50
pro	116,1	70	10	60	50
val	118,1	55	10	60	50
thr	120,1	103,2	10	60	50
ile_le	132,1	86	10	60	50
ile_le	132,1	69	10	60	50
asp	134,2	74	10	60	50
lys	147,1	84	10	60	50
glu	148,1	84	10	60	50
met	150,2	104	10	60	50
his	156,1	110	10	60	50
phe	166,1	103	10	60	50
arg	175,2	70	10	60	50
tyr	182,1	165,2	10	60	50
cys	241,2	152,1	10	60	50
trp	205	118	10	60	50
trp	205	146	10	60	50
trp	205	188	10	60	50
asn	133,1	74	10	60	50
gln	147,1	84	10	60	50

Table S5. *Viscum album* MT#39998 protein identification. The proteins identified, the number of peptides and their sequence, and the score associated with database identification relative to the MT sample are reported. The MT was run directly (MT#39998) or purified (pv#39998) through an SP column. Both data are showed.

pv#39998	Number of peptides	score	Peptides
Viscotoxin-A3 OS=Viscum album	2	55	R.NIYNACR.L<br>K.IISGSTCPSDYPK.F
Allene oxide synthase 2, chloroplastic OS=Solanum lycopersicum	2	51	K.DFVVMVSR.L<br>R.RDHVIPEFHETYTELFETLDKEMEEK.G
Putative heat shock protein 2 (Fragment) OS=Pseudotsuga menziesii	1	37	ELLSEINR
Putative UPF0496 protein 5 OS=Oryza sativa subsp. indica	1	35	R.SLSLGSLR.E
Peroxidase A2 OS=Armoracia rusticana	1	35	R.IGASLIR.L
Peroxidase 23 OS=Arabidopsis thaliana	1	29	R.GFDVIDR.M
Pentatricopeptide repeat-containing protein At1g18485 OS=Arabidopsis thaliana	1	29	K.QEFVYNELVANAFVASYAK.C
F-box protein At3g19890 OS=Arabidopsis thaliana	1	18	K.FENFIAMPFK.Y
NAD(P)H-quinone oxidoreductase subunit 5, chloroplastic OS=Morus indica	1	18	K.SQNLVLMGGLK
Terpenoid synthase 9 OS=Arabidopsis thaliana	1	15	K.DHDEMLLKFAK
FBD-associated F-box protein At4g10400 OS=Arabidopsis thaliana	1	15	R.MWVVVAVSR.Y

MT#39998	Number of peptides	score	Peptides
Calmodulin OS=Bryonia dioica	5	608	K.EAFSLFDK.D
			R.VFDKDQNGFISAAELR.H
			K.EAFSLFDKDGDGCITTK.E
			R.EADVDGDGQINYEEFVK.V
			R.EADVDGDGQINYEEFVK.V
Calmodulin OS=Triticum aestivum	4	570	R.VFDKDQDGFISAAELR.H
			K.EAFSLFDKDGDGCITTK.E
			R.EADVDGDGQINYEEFVK.V
			R.SLGQNPTEAELQDMINEVDADGNGTIDFPEFLNLMAR.K
Calmodulin-1 OS=Solanum tuberosum	3	109	K.EAFSLFDK.D
			K.VFDKDQNGFISAAELR.H
			K.EAFSLFDKDGDGCITTK.E
Viscotoxin-A3 OS=Viscum album	3	95	R.NIYNACR.L
			K.IISGSTCPSDYPK.F
			K.LSGCKIISGSTCPSDYPK.F
Viscotoxin-B (Fragment) OS=Viscum album	1	67	K.IISASTCPSDYPK.F
Ribulose bisphosphate carboxylase small chain SSU1, chloroplastic OS=Lemna gibba	2	67	K.EVDYLLR.N
			K.EGFVYRENNASPGYYDGR.Y
Ribulose bisphosphate carboxylase small chain 1A, chloroplastic OS=Arabidopsis thaliana	1	50	K.EVDYLIR.N
	1	50	R.LACGVVGLTPL.
Superoxide dismutase [Cu-Zn], chloroplastic OS=Petunia hybrida	1	50	R.LACGVVGLTPI.
	1	50	R.IGASLIR.L
Thionin OS=Pyrularia pubera	1	47	K.IISGTTCPSDYPK.-
Allene oxide synthase 2, chloroplastic OS=Solanum lycopersicum	1	47	K.DFVVMVSR.L
Viscotoxin-1-PS OS=Viscum album	1	42	K.IISASTCPSYPBK.
ABC transporter G family member 30 OS=Arabidopsis thaliana	1	42	R.GLNFGR.H
Superoxide dismutase [Cu-Zn] OS=Ananas comosus	1	34	R.VACGIIGLQG.

Beta-galactoside-specific lectin 1 OS=Viscum album	2	34	R.FITLLR.D
			R.SILILIQMISEAAR.F
Beta-galactoside-specific lectin 3 OS=Viscum album	1	30	R.FITLLR.D
Peroxidase 23 OS=Arabidopsis thaliana	1	30	R.GFDVIDR.M
Remorin 4.1 OS=Arabidopsis thaliana	2	24	K.AQRKAEER.R
			R.AKAMEKTQNK.V
Beta-galactoside-specific lectin 2 OS=Viscum album	1	19	R.FITILR.D
DEAD-box ATP-dependent RNA helicase 40 OS=Arabidopsis thaliana	1	19	K.SCVLIATDVAAR.G
Spermidine hydroxycinnamoyltransferase 1 OS=Oryza sativa subsp. japonica	2	18	.MKLDSFMVTR.R
			.MKLDSFMVTR.R
Oxygen-evolving enhancer protein 1, chloroplastic OS=Chlamydomonas reinhardtii	2	17	K.AGVRAARPNR.A
			R.AAQSAKAGVRAARPNR.A

Table S6. The mother tincture viscotoxins were analysed by HPLC as described in ref. 92.

**HPLC Quantification MT 39998**		**LC MRM MS MT 39998**
	µg/mL	µg/mL
VTA1	75,6288	115
VTA2	165,185	242
VTA3	221,442	316
VTA B	0	42,0
VT Tot	462,256	770
